# The Effect of Silver Nanoparticle Addition on the Antimicrobial Properties of Poly(methyl methacrylate) Used for Fabrication of Dental Appliances: A Systematic Review

**DOI:** 10.3390/ijms262311633

**Published:** 2025-11-30

**Authors:** Kacper Galant, Maja Podziewska, Maciej Chęciński, Kamila Chęcińska, Natalia Turosz, Dariusz Chlubek, Tomasz Korcz, Maciej Sikora

**Affiliations:** 1 Faculty of Medicine, Medical University of Lodz, Al. Kościuszki 4, 90-419 Łódź, Poland; kacpergalant.ld@gmail.com (K.G.); maja.podziewska@stud.umed.lodz.pl (M.P.); 2 National Medical Institute of the Ministry of the Interior and Administration, Wołoska 137, 02-507 Warsaw, Poland; maciej.checinski@pimmswia.gov.pl (M.C.); kamila.checinska@pimmswia.gov.pl (K.C.); natalia.turosz@pimmswia.gov.pl (N.T.); tomasz.korcz@pimmswia.gov.pl (T.K.); sikora-maciej@wp.pl (M.S.); 3 Department of Maxillofacial Surgery, Hospital of the Ministry of Interior, Wojska Polskiego 51, 25-375 Kielce, Poland; 4 Department of Biochemistry and Medical Chemistry, Pomeranian Medical University, Powstańców Wielkopolskich 72, 70-111 Szczecin, Poland

**Keywords:** silver nanoparticles, antimicrobial properties, poly(methyl methacrylate), PMMA, dental materials, nanotechnology

## Abstract

Polymethyl methacrylate (PMMA) is widely used in modern dentistry, particularly in prosthodontics, orthodontics, and maxillofacial surgery. To improve the properties of PMMA, silver nanoparticles (AgNPs) are incorporated to enhance the antibacterial, antiviral, and antifungal effects of this material. This study aims to evaluate the antimicrobial properties of AgNPs as an additive to PMMA. Medical databases covered by the ACM, BASE, PubMed, and Scopus engines were searched. Of the 670 identified records, 23 studies were included that assessed the antibacterial and antifungal properties gained by incorporating AgNPs into PMMA. All of the studies included also contained a control group—PMMA without additives. Studies that evaluated nanoparticles other than AgNPs or materials other than PMMA were excluded. The data collected from the articles included the size and concentration of the nanoparticles, the method of sample preparation, sample size, information on the effect of nanoparticles on antimicrobial properties, and the contact time between the sample and the test tube containing fungi or bacteria. The data were presented in tables and graphs. The analysis indicated that increasing the weight percent concentration of AgNPs or extending the incubation time increases the antifungal efficacy. The result of Tau Kendall correlation showed that the pairs of data, concentration/incubation time and outcomes, are inversely proportional for fungi (*p* < 0.01). The results of the study are not entirely conclusive. Some limitations suggest the need for more standardized studies, which ideally should be conducted on human research groups and followed by a study of these properties and their effects on the human body. This systematic review followed PRISMA 2020 guidelines. The protocol was submitted to the Open Science Framework Registries (1 December 2024).

## 1. Introduction

Polymethyl methacrylate (PMMA), known as acrylic, is widely used in modern dentistry as the primary material for fabricating dental appliances [1,2]. It is used for producing temporary crowns, orthodontic retainers, complete, and partial dentures, as well as for their repair. Additionally, this polymer can be used to manufacture artificial teeth for dentures [1,3]. Other applications for this material include the production of impression trays, obturators for cleft palates, printed or milled casts, treatment planning matrices, removable orthodontic appliances, and occlusal splints [1,4,5]. PMMA is also effective for the conservative treatment of fractures, ensuring proper bone positioning during orthognathic surgeries, and for treating temporomandibular joint dysfunctions [6,7,8]. Extensive acrylic prostheses and epitheses can restore facial bone defects [9].

The above-mentioned wide application of PMMA is possible thanks to its properties, including mechanical strength, low density, the possibility of individual adjustment to the patient, satisfactory esthetics, and ease of repair [10]. To further improve its characteristics, nanoparticles can be added [1], affecting the mechanical strength of the acrylic [7,10], antimicrobial activity [11,12], thermal conductivity [13], and also water sorption [14].

According to Habib et al. and Mylonas et al., PMMA can act as a reservoir for many microorganisms [15,16]. The porous surface of the acrylic appliance promotes the accumulation and formation of microbial colonies. The presence of dentures and Candida albicans can cause a chronic inflammatory reaction known as denture-induced stomatitis, which affects about 60–70% of complete denture wearers [17,18,19]. These fungal pathogens form a biofilm on the surfaces of acrylic dentures, adhering to the irregularities and rough texture [17]. This is a common problem in dentistry, which dentists are trying to combat by using antifungal and antimicrobial agents that would reduce the titer of fungal and bacterial microorganisms colonizing the denture and the mouth. After full laboratory polymerization of PMMA, there is no complete chemical reaction from the monomer to the polymer, resulting in the presence of free unreacted monomer, which increases the porosity of the prosthesis over its entire surface. This is an ideal site for colonization by viruses, bacteria, and fungi.

Prosthetic laboratories aim to reduce the porosity of the material, which affects colonization by microorganisms, by conducting the polymerization process at higher temperatures to minimize the amount of remaining monomer. They also use higher atmospheric pressure to increase the conversion of the monomer to polymer. This improves mainly mechanical properties, but microbial colonization can also be reduced by lowering porosity. Nevertheless, these surfaces are not smooth and provide an ideal niche for biofilm formation and retention, which is highly resistant to many antiseptic agents [20].

PMMA is a material that has been used in dentistry for many years, starting in 1843 [21]. However, it has both advantages and disadvantages. The solution to the problems of the PMMA is found in the addition of nanoparticles, which improve the properties of this dental material. The main nanoparticles being tested are Alumina (Al_2_O_3_), which has good biocompatibility properties [1,3]. Another additive is Zirconia (ZrO_2_), which significantly improves fracture toughness, mechanical properties, compressive and fatigue strengths [1,4]. Titania (TiO_2_) added to PMMA affects thermal conductivity, hardness, and fracture toughness. Nanodiamond (ND) addition affected PMMA elastic modulus and impact strength. Hydroxyapatite was also added, which affected the elastic modulus and flexural strength of PMMA [1].

A potential solution that could revolutionize the problem of stomatitis caused by microorganisms colonizing acrylic materials is the incorporation of silver nanoparticles (AgNPs) [17], as they demonstrate significant antimicrobial activity due to their large surface-to-volume ratio, even at low concentrations [22]. The antimicrobial activity of samples containing AgNPs is mainly an indirect effect, which occurs as a result of the release of Ag^+^ ions [23,24,25]. The released ions interact with the respiratory chain proteins of microbial cells and induce the production of reactive oxygen species, leading to oxidative stress and microorganism’s cell death [26]. They are used as an additive in surgical mesh, in producing artificial joint prostheses, and as medications to promote wound healing [27].

In modern dentistry, incorporating AgNPs enhances the quality of dental materials and improves treatment outcomes [28,29,30]. They are added to acrylic resins in prosthetic treatment, composite resins in restorative dentistry, irrigation solutions and filling materials in endodontics, and adhesive materials in orthodontics. AgNPs are also incorporated into titanium coatings that cover implants, and into membranes for guided tissue regeneration in the treatment of periodontal disease [31]. The effectiveness of AgNPs has also been proven in general dentistry (NanoCare Plus Silver Gold^®^), with proven bacteriostatic action against S. Mutans [32].

The aim of our study is to review the antifungal and antimicrobial activity of AgNPs used as an additive to PMMA.

We state the following hypothesis: the addition of AgNPs to PMMA gives antimicrobial properties, effectively inhibiting the development and growth of microorganisms, compared to acrylic without the addition of such nanoparticles.

## 2. Materials and Methods

PRISMA 2020 guidelines were used to create this review. The checklists for the report and abstract can be found in Supplementary Documents S1 and S2, respectively. The protocol was previously prospectively submitted to the Open Science Framework Registries (https://osf.io/9e6sy; 1 December 2024).

### 2.1. Eligibility Criteria

The eligibility of articles was determined by introducing appropriate PICOTS inclusion criteria: (1) Problem—acrylic blocks and all types of intraoral appliances made of PMMA with added silver nanoparticles, i.e., dentures, retainers, trays, guards and splints. (2) Intervention—the performance of any type of test to determine or estimate at least one of the following: the antimicrobial or antifungal effect of the PMMA/AgNP composites. (3) Comparison—as a control, it was necessary to test corresponding samples made of pure PMMA under the same conditions. (4) Outcome—as an outcome, it was required to report the value of reduction in the number of colony forming units (CFU) or prevention of biofilm formation on acrylic appliances or the antifungal effect for PMMA material with and without the addition of silver nanoparticles. (5) In order not to limit the search in any way and to improve its quality, no restrictions were introduced regarding publication time. (6) Study design—basic experimental studies of in vitro nature were allowed. Additionally, preprints, non-original and non-English articles, systematic reviews, etc., and other types of studies such as in vivo were excluded. A brief summary of the PICOTS criteria is presented in Table 1.

### 2.2. Information Sources

On 27 November 2024, searches were conducted in the following databases: PubMed, ACM, BASE, and Scopus (Table A1). It included publications from the databases’ inception until that date.

### 2.3. Search Strategy

Three authors (K.G., M.P. and M.C.) created the query to search for relevant articles. The search strategy is presented below:

(pmma OR polymethyl OR methacrylate OR acrylic) AND (appliance OR appliances OR device OR devices OR aligner OR aligners OR retainer OR retainers OR denture OR dentures OR prosthesis OR prostheses OR tray OR trays OR guard OR guards OR splint OR splints) AND (ag OR agnp OR silver) AND (nano OR nanoparticle OR nanoparticles) AND (antimicrobial OR microbicidal OR microbiostatic OR antibacterial OR bacteria-inhibiting OR bactericidal OR bacteriostatic OR antibiofilm OR biofilm-inhibiting OR antifungal OR fungicidal OR fungostatic OR antimycotic OR mycoticidal OR mycostatic OR antiviral OR viral-inhibiting OR virocidal OR virostatic OR antiseptic OR biocide)

Based on the presented query, a search was conducted using selected search engines (ACM, BASE, PubMed, Scopus). Scientific publications that were assessed as meeting the designated eligibility criteria were included in this review.

### 2.4. Selection Process

Using the Rayyan automation tool (version 2024.08.29; Rayyan Systems Inc., Cambridge, MA, USA), two authors (K.G. and M.P.) manually deduplicated the articles. Then, the accepted records were screened by the same two authors based on the title and abstract according to the eligibility criteria (Table 1). Cohen’s *κ* coefficient was calculated using Google Workspace software (Version 2024.08.23; Google LLC, Mountain View, CA, USA) to express inter-rater agreement. In case of discrepancies, the articles were moved to the next stage of evaluation, which consisted of a full-text review.

### 2.5. Data Collection Process

Data obtained from included articles were manually extracted into tables by two authors (K.G. and M.P.) using Google Workspace software (Version 2024.08.23; Google LLC, Mountain View, CA, USA). In case of discrepancies, the final decision was made in consultation with the third author (M.C.). Due to biological and structural differences, data on bacteria and fungi have been separated into different tables. They were then transferred into spreadsheet using Google Workspace software (Version 2024.08.23; Google LLC, Mountain View, CA, USA).

When the data were not presented in numerical form, a table, or continuous text but presented on the diagram, and when the data were difficult to read, an attempt was made to contact the author to clarify them [33].

Data were collected in which samples containing nanoparticles were matured for a maximum of seven days under controlled conditions. When samples were tested for antimicrobial properties after more than seven days, they were omitted or excluded.

### 2.6. Data Items

The following data were collected: (1) nanoparticle size and concentration, (2) sample preparation method, (3) sample size, (4) data on the effect of nanoparticles on antimicrobial properties, (5) sample contact time with sample containing fungi/bacteria. Table 2 presents the synthesized data and their definition to help the reader better understand it.

### 2.7. Study Risk of Bias Assessment

The authors assessed methodological behaviour in eligible articles using the QUIN [34]. This tool comprises twelve criteria, including clearly stated aims/objectives, detailed explanation of sample size calculation, detailed explanation of sampling technique, details of comparison group and detailed explanation of methodology. Moreover, it comprises operator details, randomization, method of measurement of outcome, outcome assessor details, blinding, statistical analysis and presentation of results. To increase the transparency of the assessment, a dedicated table was created illustrating the judgments for each included study across all domains using the QUIN tool.

### 2.8. Effect Measures and Synthesis Methods

The results of the antimicrobial properties tests were presented as a relative result. The percentage ratio of the variable value for PMMA/AgNP divided by the value of the same variable for PMMA was calculated. The data were organized into tables and visualized using charts, including trendlines where applicable. Analysis and visualization were performed using Google Sheets (Google LLC, Mountain View, CA, USA), Microsoft Excel (Microsoft Corporation, Redmond, WA, USA), and Statistica software (version 13, TIBCO Software Inc., Palo Alto, CA, USA). The regression models were analyzed: linear, quadratic, and logarithmic. The statistical validity and fit of these regression models were assessed by calculating the coefficient of determination (R^2^) and the corresponding *p*-value.

The correlation analysis was performed using the Kendall Tau test, in the form of a correlation matrix. The table was supplemented with statistical significance and the number of important conditions for 3 quantitative variables—concentration (%), incubation time (h), and antimicrobial efficacy (%). The significance level of *α* = 0.05 was assumed.

The linear trend analysis was used following a poor fit observed with other trend models and based on the hypothesis that increasing the NPs content and/or sample contact time leads to a gradual uniform increase in antimicrobial efficacy. Additional use of the Tau-Kendall test allowed for the assessment of the strength and direction of the relationship. It was chosen due to its robustness to a non-normal distribution, limited influence of outliers, and tied ranks.

## 3. Results

### 3.1. Selection of the Study

**Figure 1:**
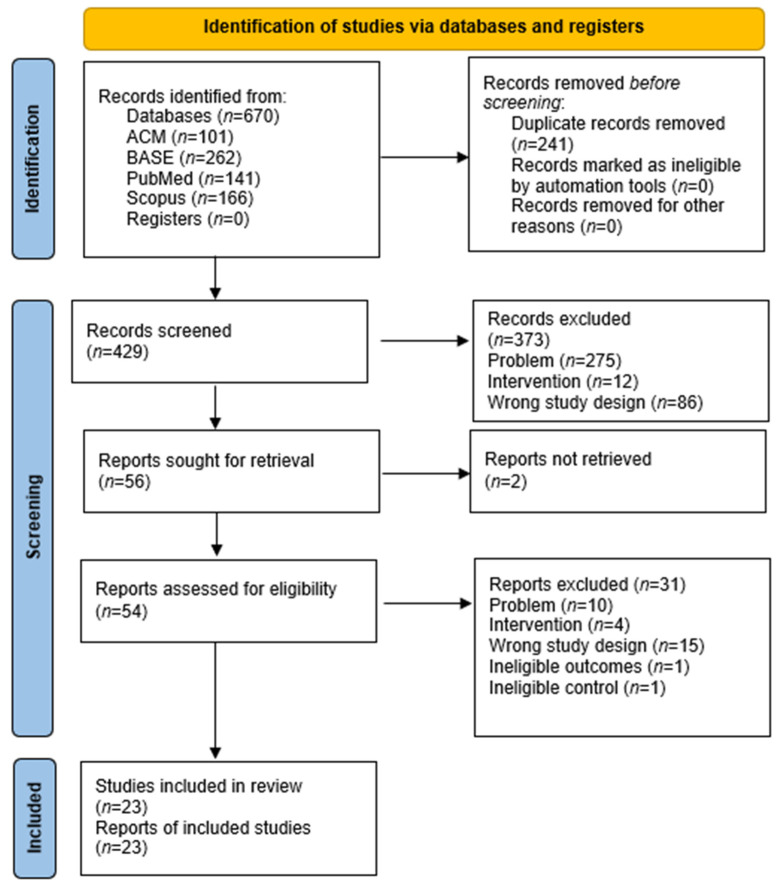
Flow diagram.

The selection process is presented in Figure 1. Of the 670 records retrieved, 241 were identified as duplicates and excluded. By evaluating the title and abstract, a total of 373 articles were excluded (275 articles due to a different research problem, 12 due to a different intervention, in addition, 86 articles had the wrong study design) with the agreement of both authors assessed at *κ* = 0.936. The number of 56 reports sought for retrieval was obtained. However, two reports were not retrieved. We assessed 54 articles to check their eligibility for inclusion in our systematic review. After full-text analysis, we excluded 31 reports from the study due to different problem, intervention, wrong study design, ineligible outcomes, and ineligible control. The number of studies included in our review was 23, while 54 reports of included studies were obtained.

### 3.2. Results of Individual Studies

Finally, results from 23 studies were included (Table 3 and Table 4). Antifungal properties were extracted from 22 studies (Table 3), while antibacterial properties were identified in 4 studies (Table 4). Detailed data from both analyses are presented in Table A2 and Table A3. Despite using the sequence regarding the antiviral property of PMMA with AgNPs in the query, no article addressing these issues was retrieved.

The samples mainly were circular in shape but had different dimensions. Nanoparticles had sizes up to 100 nm. The PMMA samples were produced using various methods, with the heat-curing method dominating the others.

In the case of the Mukai et al. article, extracting data on bacteria was impossible due to the difficult interpretation of their presentation [33].

### 3.3. Results of Syntheses

Data analysis was performed separately for fungi and bacteria. For each pair of data (Outcome (%) vs. Concentration (weight percent (wt%)); Outcome (%) vs. Incubation time (h)), a good fit of the trend line was not obtained, regardless of the selected regression model. Attempts to narrow the data in order to unify them did not bring the desired results.

**Figure 2:**
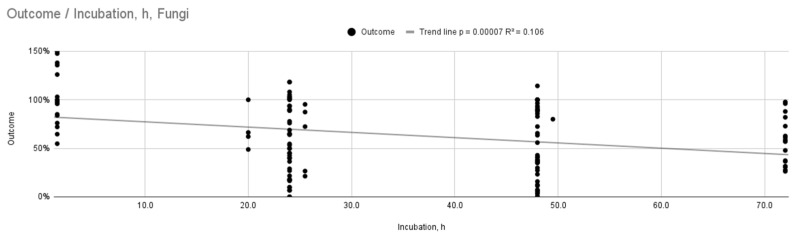
Relationship between outcome and incubation time for fungi.

**Figure 3:**
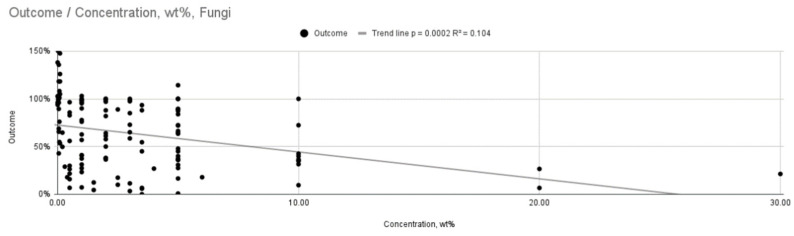
Relationship between outcome and AgNPs concentration for fungi.

Figure 2 and Figure 3 show a graph of the relationship between outcome and incubation time or wt concentration. They represent a linear regression model. It revealed statistically significant trends, but with low coefficients of determination, indicating a weak overall fit (R^2^ = 0.106, *p* = 0.00007; R^2^ = 0.104, *p* = 0.0002). The antifungal efficacy increases with increasing wt concentration of silver nanoparticles or incubation time. In the case of wt concentration, the trend line shows a steeper slope, so the concentration probably has a greater influence on the obtained result.

**Figure 4:**
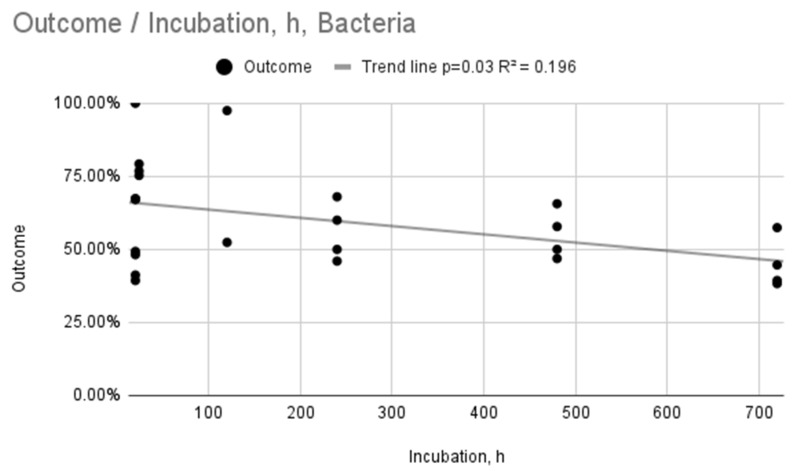
Relationship between outcome and incubation time for bacteria.

**Figure 5:**
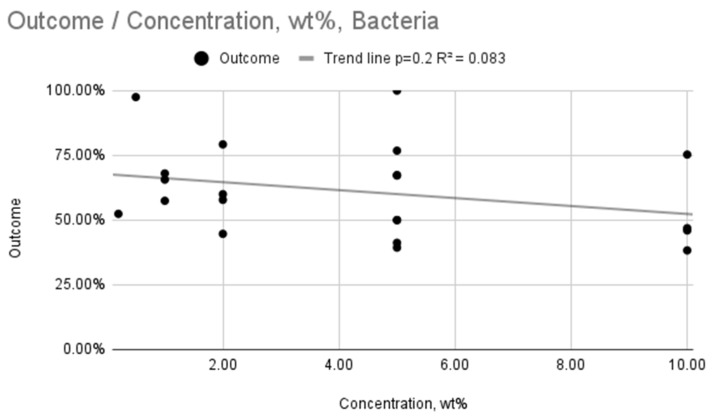
Relationship between outcome and AgNPs concentration for bacteria.

The same analysis was performed for bacteria, presented in Figure 4 and Figure 5. In the case of both relationships (Figure 4 and Figure 5), the mean practically did not change, and the trend lines did not decrease significantly, which may indicate a lack of effectiveness in a given time interval and for a given weight concentration range. Both intervals are relatively wide, in particular the incubation time (up to 720 h), so it may also result from the lack of effectiveness of AgNPs in relation to the bacteria used or their resistance to the substance used. The relationship between Outcome (%) and Incubation time (h) (Figure 4) was found to be statistically significant (*p* = 0.03; R^2^ = 0.196). In contrast, the relationship between Outcome (%) and AgNPs concentration (wt%) (Figure 5) was not statistically significant (*p* = 0.2, R^2^ = 0.083). This suggests that while longer incubation time has a weak but significant effect on antibacterial properties, the effect of AgNP concentration was not statistically discernible in the aggregated data.

Table 5 and Table 6 show the results of the Tau Kendall correlation test for fungi and bacteria, respectively. In the case of fungi, the number of valuable cases was higher (130–136). All results obtained were statistically significant for fungi (*p* < 0.05) in contrast to bacteria. For the fungi, the Tau Kendall test values indicate a weak correlation (l*τ*l < 0.3).

### 3.4. Reporting Biases

Of the twenty two included articles, two were assessed as low risk of bias, with all twelve criteria fully met. Seventenen of them were assessed as moderate risk due to concerns related to randomization, operator details, and outcome assessor details. Table 7 summarizes RoB assessments of included studies.

## 4. Discussion

### 4.1. General Interpretations of Results

In the presented systematic review, information was collected on the effect of adding silver nanoparticles to PMMA on the growth of bacteria and fungi. This effect was determined and analyzed depending on the time of contact of the sample with the microorganism and the concentration of AgNPs.

In the case of fungi, increasing the concentration of nanoparticles led to a stronger antifungal effect, as demonstrated by various research teams. This finding was also supported by studies conducted by Gopalakrishnan et al. [55], De Matteis et al. [36], and Li et al. [42] across different incubation times. These authors conducted an experiment using different concentrations and different incubation times. Ismaeil et al. [40] did not obtain such results for different incubation times—for longer incubation times, they did not obtain better results when the concentration increased. Interestingly, Suganya et al. [50] obtained the complete reduction of microorganisms, regardless of the concentration (3.5 wt% and 5.0 wt%). At this point, it is also worth noting the results obtained by Gligorijević et al. [39] and Arf et al. [35]. In their studies, opposite results were obtained—a complete lack of effectiveness against fungi and no effect. Pinheiro et al. [46] proved antimicrobial activity, but their result was the same for different concentrations used (2.5 wt% vs. 5.0 wt%).

A comparison of antimicrobial activity with incubation time is difficult because few authors performed tests for different incubation times, and the main variance was using different percentages of nanoparticles. Gopalakrishnan et al. [55] and De Matteis et al. [36] obtained better results (stronger antifungal relationship) when the sample contact time was 48 h instead of 24 h, regardless of the concentration and method used. Ismaeil et al. [40] also obtained better results (48 h vs. 72 h). Similarly, Li et al. [42] reached such a conclusion, but the incubation time was very different (1.5 h vs. 72 h).

In the case of bacteria, increased bacterial reduction was achieved with higher concentrations, as reported by Gligorijević et al. [39], and Gopalakrishnan et al. [55], and Fan et al. [37]. Antibacterial properties depending on the different sample contact times were studied only by Gopalakrishnan et al. [55]. For almost every concentration wt, increased contact time was associated with increased antibacterial activity.

Two things may have a fundamental impact on the obtained results, apart from the study design itself: (1) the mechanism of antimicrobial action—the effectiveness of which may vary depending on the species and type of bacteria/fungi, (2) the development of resistance by microorganisms—which would also require the use of a uniform group of microorganisms tested by different authors, and is impossible to do due to too many individual characteristics.

The study conducted by Hochvaldová et al. [56] presents a mechanism for the rapid development of Gram-negative and positive bacteria resistance to silver nanoparticles. This study was conducted on the following bacteria: *E. coli* and *S. aureus*. Probably, this effect is based on the fact that bacteria target the weakest point of nanoparticles, i.e., aggregation stability, which is very sensitive to environmental influences. This mechanism is most likely for both groups of bacteria because it is the simplest way to destabilize nanoparticles and eliminate their strong antibacterial properties. This effect requires only phenotypic adaptation compared to the complicated mechanisms of antibiotic resistance. In the case of Gram-negative bacteria, resistance is caused by the production of flagellin, whereas in Gram-positive bacteria, it is related to the formation of dental plaque. The good news is that it can be overcome by different strategies and, again, induce strong antibacterial properties of AgNPs.

The size of the nanoparticles used is a crucial property that directly impacts their performance. Smaller particles in the 1–10 nm range are preferred for AgNPs [57], as NPs of this size exhibit superior antimicrobial activity, as demonstrated in various studies [58,59,60]. Furthermore, as their size decreases, their specific active surface area increases [61]. This information is crucial for analyzing the data included in this review. The inconsistent results of the included primary studies also stem from the dramatic differences in the size of the nanoparticles used. The antimicrobial effect of particles in the 5–10 nm range cannot be effectively compared to that of particles in the 90–100 nm range.

Other elements that directly impact the results of the study are the physical and chemical properties of the samples. They depend on the type of material used and the manufacturing technique. As shown by Morgan et al. [62] the roughness and the type of the acrylic resin has a direct impact on the ability of bacteria to adhere and biofilm to form. In a systematic review created by Vincze et al. [63], the lowest roughness was obtained as a result of the milling technique. However, the lack of systematization of methods and using different methods of polishing the samples contribute to significant interference in the results. There are, however, studies with opposite conclusions, in which the hypothesis that there is a correlation between the roughness of the surfaces of various dental materials, not only PMMA, that has an influence on bacterial adhesion, has not been confirmed [64].

### 4.2. Strengths

The strengths of this review include a very broad query without time constraints, which made it possible to obtain an impressive number of articles we analyzed from many databases. A large number of articles were included in our review. While creating the review, we focused on a limited subject matter, which resulted in a very specific publication. The antimicrobial properties we analyzed include the antibacterial and antifungal activities of silver nanoparticles, which contribute to the high quality of the studies presented in the context of the activities of AgNP additives to PMMA.

The data collected in this review certainly points to a strength: adding AgNPs to PMMA shows potential antimicrobial effects in a lab setting. This is a promising starting point for developing better dental materials.

However, we must be very clear about the limits of this in vitro data. What happens in a test tube is not the same as what happens in a patient’s mouth. Therefore, the idea that this material is a ready-made ‘solution’ for clinics or that dentists ‘can introduce’ it into their practice is simply not supported by the evidence. Such statements are too optimistic and overlook the crucial steps of clinical validation.

Before we can suggest any clinical use, we absolutely need in vivo studies to prove that this material is both safe and effective in the complex oral environment. Right now, any recommendation for patient use based only on these findings would be scientifically unfounded.

Eliminating microorganisms from the oral cavity, especially fungi, is vital, as it has inconvenient consequences for denture wearers. The symptoms of a fungal infection can be very different and lead to many complications of varying severity. For patients, colonization of the denture base by microorganisms clinically manifests itself starting with the most common symptoms such as redness, burning sensation, dryness, inflammation of the mucosa, inflammation of the hard palate, erythematous inflammation, angular cheilitis, and median rhomboid glossitis [20,65,66]. It can even lead to life-threatening aspirational pneumonia [67]. The treatment of such inflammation is burdensome and prolonged. Topical treatment with antifungal drugs such as nystatin and amphotericin B is used. In addition, antiseptics and disinfecting agents are recommended. In other cases, it may be necessary to join systemic treatment, which is also associated with side effects. In addition, after each fungal infection, the prosthesis should be redone, and even so, we have no assurance that the new prosthesis will not be re-colonized with microorganisms and the problem will reappear [68,69].

Our updated analysis, including the latest research, reinforces the value and significance of the results presented in our review. In addition, our review has identified areas that require further research, which may inspire further projects and studies that will develop the topic we have covered.

### 4.3. Limitations

In the case of this review, the data were characterized by a high degree of diversity, which made their synthesis difficult.

First of all, the authors studied different species of fungi and bacteria. There was little variability in fungi, because *C. albicans* dominated and only Souzna Neto et al. tested *C. glabrata*. However, in the case of bacteria, there was a greater diversity. *S. mutans* dominated, but other bacteria, such as *S. aureus* and *E. coli*, also appeared. This makes it difficult to evaluate the obtained data because the species are characterized by different degrees of virulence, resistance, etc. The availability of data for only a few selected microbial species is a significant limitation of the available literature. Although these species are of significant clinical importance, they do not represent the polymicrobial nature of the oral microbiome. This is one important finding indicating a research gap in the available literature. Furthermore, attempting to combine diverse microorganisms would be statistically weak. Paradoxically, the current literature is both too narrow (lack of species diversity) and too heterogeneous (biological diversity) to conduct a reliable statistical synthesis.

In addition, the authors did not use the same type of microbiological medium during the experiments, and the samples were characterized by different concentrations of bacteria/fungi.

The shape and size of the samples were also not constant. This causes an increase or decrease in the total sample volume and a simultaneous increase or decrease in the total content of nanoparticles, which induces an antimicrobial effect. In addition to the mentioned shape and size, the total content of nanoparticles is also influenced by the different percentages of silver nanoparticles, which were variable. It is worth emphasizing again that the antimicrobial activity of modified PMMA also depends on the size of the NPs used. The wide range of their size limits the ability to perform effective statistical analysis. This lack of precise material characterization is a major flaw in the current body of literature.

Also, the measurement method used was not uniform and varied between the authors. Since these methods often examine different aspects of antimicrobial activity, the results were diverse with varying methods of measurement, even with the same author.

The samples made of PMMA were also subjected to different maturation times. To systematize this variable, and at the same time not to exclude too many publications, this period was narrowed to 7 days. However, this time, it could dramatically affect the properties of the samples and change them.

Often, authors present data in the form of a bar chart, in which approximate values were read and marked in Table 4 and Table 5. Unfortunately, some data could not be read due to their difficult interpretation.

In our analysis, we only considered data on AgNP concentration and contact time of the sample with microorganisms. In this way, we eliminated analysis errors, although there should be no differences in the other variables in an ideal synthesis.

In addition, most of the included articles showed medium Risk of Bias, which may indirectly affect the statistical power of the analyses performed.

### 4.4. Applicability and Future Perspectives

Our publication indicates gaps in the available literature. PMMA is still a very popular material despite many years on the market, and there is still a lack of its worthy equivalent. This is why various studies and interventions are important to maximize the valuable features and improve this material. The microbiome in the oral cavity is also inextricably linked to the use of PMMA. It consists of over 700 species of bacteria, fungi, viruses, and archaea [70]. Hence, the constant search for a golden mean as a response to the risk of infection with opportunistic fungi or other aggressive microorganisms associated with the use of PMMA is needed. The introduction of modifications of PMMA with various additives like fluoride glass fillers [71,72], fluorapatite, or apatite-coated TiO_2_ [11,12], nanodiamonds [73], and mesoporous silica nanoparticles loaded with the antifungal medicament amphotericin B [74] give hope for solving this problem.

As shown by Galant et al. [4] in a review on the effect of AgNP addition on mechanical properties, there is a range of nanoparticle sizes and concentrations in which optimal flexural, impact, and tensile strength is maintained (15–70 nm and a concentration of 0.5–4.0 wt%). This author indicated specific theoretical most optimal peak values in which these properties will be maintained.

As shown by Chęcińska et al. [7], there are other nanoparticles that can improve the mechanical properties (Flexural strength, impact strength, tensile strength) of dental devices made of acrylic resin—ZrO_2_ nanoparticles.

The future of such popular and often chosen devices made of PMMA may depend on the proper selection and configuration of nano additives. The current lack of a better equivalent, and if it occurs, the long time to market may be crucial for developing such a sector of interest. Further research is needed to explore the antimicrobial properties of silver nanoparticles (AgNPs) at concentrations that maintain their effectiveness while also preserving the mechanical properties of the material. Additionally, incorporating at the same time other nanoparticles, such as zirconium dioxide (ZrO_2_), may enhance these mechanical properties. Such connections would translate into resistance to the enormous forces occurring in the patient’s oral cavity, as well as those arising in the event of dropping or crushing the prosthesis outside of the mouth. Such advancements would help address the main disadvantages of PMMA. However, for this to happen, future studies must strive to standardize research protocols (e.g., standardized sample preparation methods, common measurement methods, etc.). The use of multispecies biofilm models, which are more representative of the oral environment, would further enrich the research. Only in this way will it be possible to conduct a valuable meta-analysis. Before moving on to in vivo studies, it is necessary to examine the long-term stability of the modified materials. It is necessary to verify that the released Ag+ ions do not exceed toxic levels and to examine the cytotoxicity of this type of PMMA against human oral cells (gingival fibroblasts, epithelial cells). Studies show that the toxicity of AgNPs is dose-dependent and can cause damage to distant organs. Furthermore, despite doses below the LOAEL, long-term, repeated exposure to AgNPs at low doses can still induce damage and pathology in related organs [75]. The toxicity is primarily due to the same mechanism as their antimicrobial activity—it is primarily caused by the formation of ROS, which can damage the cell membrane and lead to cell apoptosis [76,77].

## 5. Conclusions

Based on 23 studies, it is not possible to draw clear conclusions regarding the antimicrobial properties of silver nanoparticles. The significance of this study lies in the finding that while the addition of AgNPs demonstrates a statistically significant, albeit weak, trend in antimicrobial activity against key pathogens, the overall field of research is limited by two main factors: a lack of methodological standardization and a narrow focus on just a few microbial species. While the current trend indicates the potential of modified PMMA, the road to clinical application is still long. Several challenges must be addressed to bridge the gap between laboratory potential and a viable biomaterial (i.e., commercialization). Further studies are needed to unify the results by using standardized methods. It is necessary to assess the toxicity of these nanoparticles and their impact on the human body before conducting in vivo studies using human research groups. It must be strongly emphasized that all findings in this review are based on in vitro studies. These laboratory results cannot be directly translated to the complex clinical reality of the oral environment, and no conclusions regarding patient safety or clinical efficacy can be drawn at this stage.

## Figures and Tables

**Table 1:** Eligibility Criteria.

	Inclusion Criteria	Exclusion Criteria
Problem	Intraoral dental appliances made of PMMA with AgNPs	The additive of other types of nanoparticles, i.e., zircon, other materials than PMMA, not dental appliances
Intervention	Test of antibacterial or antifungal properties	Test performed more than 7 days after fabrication
Comparison	PMMA without the addition of AgNPs (control group)	Control sample prepared using a method different from the test sample
Outcome	Reduction in the number of CFU, biofilm formation, or antifungal effect etc.	Non-quantitative outcomes
Timeframe	No limit	Preprints
Study design	Primary studies; in vitro studies	Non-original/non-English research, Reviews, book chapters, or book fragments

**Table 2:** Definition of included data.

Data Item	Description
Specimen dimension	Dimensions of the test sample and shape of it
Fabrication Method	Method of producing a PMMA sample
Tested bacteria/fungi	Specimen of bacteria or fungi used in testing
Sample contact time	Contact time of the sample with the bacterial/fungal suspension
AgNP size	Mean or range of nanoparticle diameter
Method of assessment	Method of assessing antimicrobial properties
AgNP concentration	Number of nanoparticles in PMMA sample, percentage by weight
Result fungi	Test results obtained for a sample containing fungi
Result bacteria	Test results obtained for a sample containing bacteria

**Table 3:** Summary of Included Studies and Key Parameter.

Microorganism Category	Primary Species Tested	No. of Studies	AgNP Concentration Range (wt%)	Incubation Time Range (h)
Fungi	*C. albicans* (21 studies), *C. glabrata* (1 study)	22	0.003% to 30%	1.5 h to 72 h
Bacteria	*S. mutans* (3 studies), *S. aureus* (1 study), *E. coli* (1 study)	4	0.2% to 10%	20 h to 720 h

**Table 4:** Summary of Antimicrobial Effects Based on Regression Analysis.

Microorganism	Analyzed Variable	No. of Studies (n)	Regression Analysis Outcome (Conclusion)
Fungi	AgNP Concentration (wt%)	22	Weak, statistically significant relationship (*R*^2^ = 0.104, *p* < 0.001)
Fungi	Incubation Time (h)	22	Weak, statistically significant relationship (*R*^2^ = 0.106, *p* < 0.001)
Bacteria	AgNP Concentration (wt%)	4	Weak, statistically insignificant relationship (*R*^2^ = 0.083, *p* = 0.2)
Bacteria	Incubation Time (h)	4	Weak, statistically significant relationship (*R*^2^ = 0.196, *p* = 0.03)

**Table 5:** Tau Kendall test for fungi.

Pair of Variables	Number of Variables (*n*)	Tau Kendall (*τ*)	Significance Level (*p*)
incubation time, h vs. concentration, wt%	130	0.181	0.002
incubation time, h vs. outcome	136	–0.237	0.00004
concentration, wt% vs. outcome	130	–0.272	0.000004

**Table 6:** Tau Kendall test for bacteria.

Pair of Variables	Number of Variables (*n*)	Tau Kendall (*τ*)	Significance Level (*p*)
incubation time, h vs. concentration, wt%	21	–0.063	0.689
incubation time, h vs. outcome	25	–0.267	0.061
concentration, wt% vs. outcome	21	–0.241	0.127

**Table 7:** Risk of bias assessment of included studies.

Author, Year	D1	D2	D3	D4	D5	D6	D7	D8	D9	D10	D11	D12	Overall
Acosta-Torres, 2012 [34]	2	0	N/A	2	2	0	0	2	0	0	2	2	Moderate
Arf, 2024 [35]	2	0	1	2	2	0	0	2	0	0	2	2	Moderate
De Matteis, 2019 [36]	2	0	N/A	2	2	0	0	2	0	0	1	2	Moderate
Fan, 2011 [37]	2	0	N/A	2	2	0	0	2	0	0	1	2	Moderate
Gad, 2022 [38]	2	0	N/A	2	2	2	0	2	0	0	2	2	Low
Gligorijevic, 2017 [39]	2	0	N/A	1	1	0	0	2	0	0	0	2	High
Ismaeil, 2023 [40]	2	0	N/A	2	2	0	1	2	0	0	2	2	Moderate
Nam, 2012 [41]	2	0	N/A	2	2	0	0	2	0	0	1	2	Moderate
Li, 2016 [42]	2	0	N/A	2	2	0	0	2	0	0	2	2	Moderate
Anaraki, 2017 [43]	2	0	N/A	2	2	0	0	2	0	0	1	2	Moderate
Peter, 2023 [44]	2	0	N/A	2	2	0	0	2	0	0	2	2	Moderate
Mukai, 2023 [33]	2	0	N/A	2	2	0	1	2	0	0	1	2	Moderate
Palaskar, 2024 [45]	2	0	N/A	2	2	0	0	2	2	0	2	2	Low
Pinheiro, 2021 [46]	2	0	N/A	2	2	0	0	2	0	0	2	2	Moderate
Sato, 2018 [47]	2	0	N/A	2	2	0	0	2	0	0	2	2	Moderate
Sonkol, 2024 [48]	2	0	1	2	1	0	0	2	0	0	0	2	High
Souza Neto, 2018 [49]	2	0	N/A	2	2	0	0	2	0	0	2	2	Moderate
Suganya, 2014 [50]	2	0	N/A	2	1	0	0	1	0	0	0	2	High
Sun, 2021 [51]	2	1	N/A	2	2	0	1	2	0	0	2	2	Moderate
Thabet, 2022 [52]	2	0	N/A	2	2	0	0	2	0	0	1	2	Moderate
Vaiyshnavi, 2022 [53]	2	0	N/A	2	2	0	0	2	0	0	2	2	Moderate
Wady, 2012 [54]	2	0	N/A	2	2	1	1	2	0	0	2	2	Moderate

## Data Availability

The original contributions presented in this study are included in the article/supplementary material. Further inquiries can be directed to the corresponding author.

## Supplementary Materials

The following supporting information can be downloaded at: https://www.mdpi.com/article/10.3390/ijms262311633/s1. Reference [78] is cited in the supplementary material.

## Abbreviations

The following abbreviations are used in this manuscript:

AgNPs	silver nano particles
N/A	not applicable
ND	Nanodiamond
PMMA	Poly(methyl methacrylate)
wt%	weight percent

## Appendix A

**Table A1:** Search engine scopes.

Engine	Scope, Records
ACM	3,797,063
BASE	414,202,196
PubMed	over 37,000,000
Scopus	over 97,300,000

**Table A2:** Data obtained from antifungal studies.

First Author, Publication Year	DOI	Specimen Dimension	Fabrication Method	Tested Fungi	Sample Contact Time(h)	AgNP Size, nm (Range, Mean)	Method of Assessment	AgNP Concentration, wt%	Result Fungi	**Antifungal Properties, % ***
Acosta-Torres, 2012 [34]	10.2147/IJN.S32391	Circle 20 × 2 mm	microwave irradiation	*C. albicans*	24	10–20 N/A	adherence; luminescent microbial cell viability assay (light relative units × 103)	N/A control	4.8 11.5	41.74
Arf, 2024 [35]	10.24017/science.2024.1.6	Circle 10 × 2 mm	cold cure acrylic	*C. albicans*	48	N/A 20	anti-adherence assessment	1.0 3.0 5.0 control	82 10 2.4 300 (CFU × 10^4^/mL)	27.33 3.33 0.8
		Circle 10 × 2 mm	cold cure acrylic	*C. albicans*	48	N/A 20	agar disc diffusion	1.0 3.0 5.0 control	0 0 0 (no effect)	0 0 0
De Matteis, 2019 [36]	10.3390/ijms20194691	N/A	heat-polymerized	*C. albicans*	24	17–23 20	enumeration of the colony-forming units (CFU × 10^6^/mL)	3.0 3.5 control	2.6 1.8 4	65 45
		N/A	heat-polymerized	*C. albicans*	48	17–23 20	enumeration of the colony-forming units (CFU × 10^6^/mL)	3.0 3.5 control	†0.8 0.41 7	11.43 5.86
		N/A	heat-polymerized	*C. albicans*	24	17–23 20	anti-adherence test (%)	3.5 control	36 66	54.55
		N/A	heat-polymerized	*C. albicans*	48	17–23 20	anti-adherence test (%)	3.5 control	6 90	6.67
		N/A	heat-polymerized	*C. albicans*	24	17–23 20	circularity	3.5 control	0.85 0.91	93.41
		N/A	heat-polymerized	*C. albicans*	48	17–23 20	circularity	3.5 control	0.81 0.92	88.04
Gad, 2022 [38]	10.1016/j.prosdent.2020.08.021	Circle 15 × 2 mm	heat-polymerized	*C. albicans*	48	5–60 20	anti-adherence assessment (direct method)	0.5 1.0 1.5 control (CFU/mL)	322.4 147.2 91.4 2035.9	15.84 7.23 4.49
		Circle 15 × 2 mm	heat-polymerized	*C. albicans*	48	5–60 20	anti-adherence assessment (slide count)	0.5 1.0 1.5 control (CFU/mL)	1075.6 838.6 446.3 3602.0	29.86 23.28 12.39
Gligorijević, 2017 [39]	10.5937/asn1775696G	Circle 10 × N/A mm	cold polymerized	*C. albicans*	48	N/A N/A	disc diffusion method	2 5 10 control	10 10 10 10	0 0 0
Gopalakrishnan, 2019 [55]	10.1080/00914037.2018.1552863	N/A	compression moulding technique	*C. albicans*	24	0–100 45	anti-candidal assay before sonication	1 2 5 10 control CFU	† 1800 † 1000 † 900 † 850 † 2000	90 50 45 42.5
		N/A	compression moulding technique	*C. albicans*	48	0–100 45	anti-candidal assay before sonication	1 2 5 10 control CFU	† 2050 † 1900 † 1800 † 1750 † 5000	41 38 36 35
		N/A	compression moulding technique	*C. albicans*	72	0–100 45	anti-candidal assay before sonication	1 2 5 10 control CFU	† 3700 † 3750 † 2000 † 2050 † 6500	56.92 57.69 30.77 31.54
		N/A	compression moulding technique	*C. albicans*	24	0–100 45	anti-candidal assay after sonication	1 2 5 10 control CFU	† 1900 † 1600 † 1000 † 1000 † 2500	76 64 40 40
		N/A	compression moulding technique	*C. albicans*	48	0–100 45	anti-candidal assay after sonication	1 2 5 10 control CFU	† 2200 † 2050 † 1950 † 1900 † 5400	40.74 37.96 36.11 35.19
		N/A	compression moulding technique	*C. albicans*	72	0–100 45	anti-candidal assay after sonication	1 2 5 10 control CFU	† 4200 † 4100 † 2500 † 2450 † 6700	62.69 61.19 37.31 36.57
Ismaeil, 2023 [40]	10.4103/jioh.jioh_62_22	Circle 10 × 2 mm	heat-polymerized	*C. albicans*	48	N/A 40	agar disc diffusion	0.5 1.0 control	17.9 26.7 10	179.0 267.0
		Circle 10 × 2 mm	heat-polymerized	*C. albicans*	72	N/A 40	elution	0.5 1.0 control (CFUs/mL)	1.50 1.78 5.70	26.32 31.23
Nam, 2012 [41]	10.1111/j.1741-2358.2011.00489.x	Circle 20 × 3 mm	wet–heat poly-merisation	*C. albicans*	25.5	N/A N/A	exposure to bacterial suspension	1 5 10 20 30 control	362 332 275 101 81 380	95.26 87.37 72.37 26.58 21.32
Li, 2016 [42]	10.1111/ger.12142	Circle 15 × 1 mm	heat-polymerized resin	*C. albicans*	1.5	N/A N/A	adhesion TFA(×104)	1 2 3 5 control	5.82 5.65 5.56 4.07 5.65	103.01 100 98.41 72.04
		Circle 15 × 1 mm	heat-polymerized resin	*C. albicans*	1.5	N/A N/A	adhesion live fungal cell percentage(%)	1 2 3 5 control	97.41 96.70 95.72 82.37 98.19	99.21 98.48 97.48 83.89
		Circle 15 × 1 mm	heat-polymerized resin	*C. albicans*	1.5	N/A N/A	adhesion the generation rate of germtubes (%)	1 2 3 5 control	91.55 90.48 79.33 60.26 93.31	98.11 96.97 85.02 64.58
		Circle 15 × 1 mm	heat-polymerized resin	*C. albicans*	72	N/A N/A	biofilm development Thickness of biofilm (um)	1 2 3 5 control	21.67 18.17 16.15 6.10 22.17	97.74 81.96 72.85 27.51
		Circle 15 × 1 mm	heat-polymerized resin	*C. albicans*	72	N/A N/A	biofilm development Live fungal cell percentage (%)	1 2 3 5 control	87.46 80.09 53.31 43.57 91.05	96.06 87.96 58.55 47.85
Anaraki, 2017 [43]	10.15171/PS.2017.31	Circle 10 × 4 mm	heat-polymerized	*C. albicans*	24	N/A 20	biofilm formation (CFU)	0.5 2.5 5 10 20 control	127 102 97 56 39 586	21.67 17.41 16.55 9.56 6.66
Peter, 2023 [44]	10.4103/jmau.jmau_58_23	Circle 5 × 1 mm	heat-polymerized	*C. albicans*	24	10–20 N/A	agar disc diffusion	2 4 6 control	13.71 18.58 27.96 5	274.2 371.6 559.2
Mukai, 2023 [33]	10.4317/jced.59766	Block 10 × 10 × 3 mm	heat polymerized	*C. albicans*	49.5	N/A N/A	anti-adherence assessment CFU × 105	N/A control	† 17,5 † 14	125
Palaskar, 2024 [45]	10.4103/jips.jips_423_23	Circle 10 × 3 mm	heat-polymerized	*C. albicans*	24	0–10 N/A	anti-adherence assessment	0.1 0.2 0.3 0.4 0.5 control	78.66 73.33 42.66 26.33 10 147.5	53.33 49.72 28.92 17.85 6.78
Pinheiro, 2021 [46]	10.1590/1980-5373-MR-2020-0115	Circle 15 × 2 mm	heat-polymerized	*C. albicans*	24	N/A 50	XTT assay protocol	1.0 2.5 5.0 control (spectrofotometer and formazon)	0.07 0.08 0.08 0.09	77.78 88.89 88.89
Sato, 2018 [47]	10.14295/bds.2018.v21i1.1534	Circle 5 × 2 mm	heat-polymerized	*C. albicans*	48	N/A	biofilm formation (CFU)	N/A N/A control	4.95 5.05 5.4	91.67 93.52
Sonkol, 2024 [48]	DOI: 10.4103/tdj.tdj_30_23	Circle 15 × 3 mm	conventional method	*C. albicans*	48	N/A N/A	agar disc diffusion	5 control	16.8 15	112.0
Souza Neto, 2019 [49]	10.1016/j.eurpolymj.2018.10.009	Block 60 × 10 × 3 mm	heat-polymerized	*C. glabrata*	48	3–13 6.7	total biofilm biomass(CV)	0.05 0.5 5.0 control	† 0.19 † 0.24 † 0.21 † 0.29	65.52 82.76 72.41
		Block 60 × 10 × 3 mm	heat-polymerized	*C. glabrata*	48	3–13 6.7	metabolic activity (XTT)	0.05 0.5 5.0 control	† 0.03 † 0.06 † 0.08 † 0.07	42.86 85.71 114.29
		Block 60 × 10 × 3 mm	heat-polymerized	*C. glabrata*	48	3–13 6.7	quantification of biofilm cultivable cells log10 CFU/cm2	0.05 0.5 5.0 control	† 5.1 † 5.5 † 5.1 † 5.7	89.47 96.49 89.47
Suganya, 2014 [50]	10.4103/0970-9290.135923	Circle 5 × 1 mm	heat polymerized acrylic resin	*C. albicans*	24	10–20 N/A	enumeration of the colony- forming units(CFU/mL)	2.5 3.5 5.0 control	10^4^ 10^2^ 10 10^5^	10 0.1 0.01
Sun, 2021 [51]	10.4012/dmj.2020-129	Circle 10 × 2 mm	N/A	*C. albicans*	20	40–60 N/A	agar disc diffusion (after 0 days)	powder 5% Solution N/A control	15.08 20.47 10	150.8 204.7
		Circle 10 × 2 mm	N/A	*C. albicans*	20	40–60 N/A	agar disc diffusion (after 7 days)	powder 5% Solution N/A control	10 16.1 10	100 161
Thabet, 2022 [52]	10.21608/edj.2022.111973.1919	Circle 15 × 2 mm	heat-polymerized	*C. albicans*	48	0–45 N/A	anti-adherence assessment (direct culture, serial dilution)	5.0 control (CFUs)	32.44 51.12	63.46
Vaiyshnavi, 2022 [53]	10.4103/jips.jips_574_21	Circle 50 × 1 mm	high-impact injection- moulded	*C. albicans*	24	N/A N/A	agar disc diffusion	0.05 0.2 control (MM)	† 72.7 † 77.4 50	145.4 154.8
Wady, 2012 [54]	10.1111/j.1365-2672.2012.05293.x	Circle 13.8 × 2 mm	microwave irradiation	*C. albicans*	1.5	N/A 9	XTT adherence assay (after 0 days storage)	0.003 0.025 0.05 0.075 0.1 control	† 0.48 † 0.49 † 0.48 † 0.38 † 0.63 † 0.5	† 96.0 † 98.0 † 96.0 † 76.0 † 126.0
		Circle 13.8 × 2 mm	microwave irradiation	*C. albicans*	1.5	N/A 9	XTT adherence assay (after 7 days storage)	0.003 0.025 0.05 0.075 0.1 control	† 0.58 † 0.63 † 0.57 † 0.23 † 0.62 † 0.42	† 138.1 † 150.0 † 135.71 † 54.76 † 147.62
		Circle 13.8 × 2 mm	microwave irradiation	*C. albicans*	24	N/A 9	XTT biofilm assay (after 0 days storage)	0.003 0.025 0.05 0.075 0.1 control	† 1.5 † 1.65 † 1.6 † 1.62 † 1.68 † 1.6	† 93.75 † 103.13 † 100 † 101.25 † 105
		Circle 13.8 × 2 mm	microwave irradiation	*C. albicans*	24	N/A 9	XTT biofilm assay (after 7 days storage)	0.003 0.025 0.05 0.075 0.1 control	† 1.52 † 1.49 † 1.75 † 1.6 † 1.75 † 1.48	† 102.70 † 100.68 † 118.24 † 108.11 † 118.24

N/A—not applicable; *—relative to the reference material; †—approximate value.

**Table A3:** Data obtained from antibacterial studies.

First Author, Publication Year	DOI	Specimen Dimension	Fabrication Method	Tested Bacteria	Sample Contact Time (h)	AgNP Size, nm (Range, Mean)	Method of Assessment	AgNP Concentration, wt%	Result Bacteria	Antibacterial Properties, % *
Fan, 2011 [37]	10.1016/j.dental.2010.11.008	Circle 9.53 × 1.59 mm	chemical cured acrylic resin	*Streptococcus mutans*	120	2–18 N/A	growth inhibition	0.2 0.5 control (%)	52.4 97.5 0	52.4 97.5
Gligorijević, 2017 [39]	10.5937/asn1775696G	Circle 10 × N/A mm	cold polymerized	*Staphylococcus aureus*	24	N/A N/A	disc diffusion method	2 5 10 control	12.62 13.01 13.28 10.00	126.2 130.1 132.8
Gopalakrishnan, 2019 [55]	10.1080/00914037.2018.1552863	N/A	compression moulding technique	*Streptococcus mutans*	240	0–100 45	anti-biofilm assay (cells)	1 2 5 10 control	† 1700 † 1500 † 1250 † 1150 † 2500	† 68.0 † 60.0 † 50.0 † 46.0
		N/A	compression moulding technique	*Streptococcus mutans*	480	0–100 45	anti-biofilm assay (cells)	1 2 5 10 control	† 2100 † 1850 † 1600 † 1500 † 3200	† 65.63 † 57.81 † 50.0 † 46.88
		N/A	compression moulding technique	*Streptococcus mutans*	720	0–100 45	anti-biofilm assay (cells)	1 2 5 10 control	† 2700 † 2100 † 1850 † 1800 † 4700	† 57.45 † 44.68 † 39.36 † 38.30
Sun, 2021 [51]	10.4012/dmj.2020-129	Circle 10 × 2 mm	N/A	*Escherichia coli*	20	40–60 N/A	agar disc diffusion (after 0 days)	powder 5% solution N/A control	14.88 20.2 10	148.8 202.0
		Circle 10 × 2 mm	N/A	*Streptococcus mutans*	20	40–60 N/A	agar disc diffusion (after 0 days)	powder 5% solution N/A control	24.26 25.4 10	242.6 254.0
		Circle 10 × 2 mm	N/A	*Escherichia coli*	20	40–60 N/A	agar disc diffusion (after 7 days)	powder 5% solution N/A control	10 14.9 10	100.0 149.0
		Circle 10 × 2 mm	N/A	*Streptococcus mutans*	20	40–60 N/A	agar disc diffusion (after 7 days)	powder 5% solution N/A control	14.83 20.73 10	148.3 207.3

N/A—not applicable; *—relative to the reference material; †—approximate value.
